# *In Vivo* Osteogenic Differentiation of Human Embryoid Bodies in an Injectable *in Situ*-Forming Hydrogel

**DOI:** 10.3390/ma6072978

**Published:** 2013-07-17

**Authors:** Da Yeon Kim, Yoon Young Kim, Hai Bang Lee, Shin Yong Moon, Seung-Yup Ku, Moon Suk Kim

**Affiliations:** 1 Department of Molecular Science and Technology, Ajou University, Suwon 443-749, Korea; E-Mails: dayeon@ajou.ac.kr (D.Y.K.); hblee@ajou.ac.kr (H.B.L.); 2 Institute of Reproductive Medicine and Population, Medical Research Center, Seoul National University, Seoul 110-810, Korea; E-Mails: yoonykim@snu.ac.kr (Y.Y.K.); shmoon@snu.ac.kr (S.Y.M.); 3 Department of Obstetrics and Gynecology, College of Medicine, Seoul National University, Seoul 110-744, Korea

**Keywords:** human embryoid bodies, *in situ*-forming hydrogel, osteogenic differentiation, tissue engineering

## Abstract

In this study, we examined the *in vivo* osteogenic differentiation of human embryoid bodies (hEBs) by using an injectable *in situ*-forming hydrogel. A solution containing MPEG-*b*-(polycaprolactone-*ran*-polylactide) (MCL) and hEBs was easily prepared at room temperature. The MCL solution with hEBs and osteogenic factors was injected into nude mice and developed into *in situ*-forming hydrogels at the injection sites; these hydrogels maintained their shape even after 12 weeks *in vivo*, thereby indicating that the *in situ*-forming MCL hydrogel was a suitable scaffold for hEBs. The *in vivo* osteogenic differentiation was observed only in the *in situ* gel-forming MCL hydrogel in the presence of hEBs and osteogenic factors. In conclusion, this preliminary study suggests that hEBs and osteogenic factors embedded in an *in situ*-forming MCL hydrogel may provide numerous benefits as a noninvasive alternative for allogeneic tissue engineering applications.

## 1. Introduction

Tissue engineering is a promising method for regenerating damaged tissues or organs [1] and requires the presence of suitable three-dimensional scaffolds [2,3,4,5,6,7,8]. Great progress has been made in the fabrication of biocompatible scaffolds, although it is prohibitively difficult to fabricate certain complicated scaffold geometries. To overcome this problem, the use of *in situ*-forming hydrogels as scaffolds is a promising approach for the fabrication of complicated scaffold geometries [9].

*In situ*-forming hydrogels are based on the concept that under physiological conditions, certain biomaterials form desired hydrogels *in situ* after injection as a liquid via a simple liquid-to-gel phase transition [10]. This characteristic enables hydrogels to easily trap various biologic materials such as growth factors, genes and cells. Many *in situ* hydrogel-forming biomaterials have been proposed, including collagen, chitosan, hyaluronate, fibrin, pluronic and various block copolymers [11,12,13,14,15,16,17].

Recently, our group reported that biodegradable methoxypoly(ethylene glycol)-b-polycaprolactone (MP) and MPEG-*b*-(polycaprolactone-*ran*-polylactide) (MCL) are potential *in situ*-forming hydrogels [18,19,20,21,22,23,24,25,26,27,28]. MCL exhibited a biodegradation window that is adjustable from a few weeks to a few months [20,21,22]. Subsequent studies have demonstrated the potential of injectable *in situ*-forming MP hydrogels as *in vitro* and *in vivo* bone graft material for rat bone marrow mesenchymal stem cells and human adipose tissue-derived stem cells [27,28].

Embryonic stem cells (ESCs) possess the property of indefinite self-renewal and are derived from the inner cell mass of preimplantation embryos at the blastocyst stage [29]. Generally, the initiation of differentiation of ESCs occurs through an intermediate step involving the formation of embryoid bodies (EBs) via complex three-dimensional aggregates of ESCs [30,31]. ESCs within the aggregated EBs can undergo differentiation into all the somatic cell types, namely, the endoderm, ectoderm and mesoderm lineages [32]. Thus, EBs have the potential to serve as an omnipotent cell resource for cell therapy, including tissue engineering.

**Figure 1:**
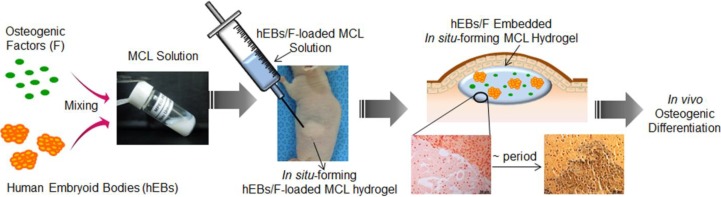
Schematic representation of *in vivo* osteogenic differentiation of human embryoid bodies (hEBs) in an injectable *in situ*-forming MPEG-*b*-(polycaprolactone-*ran*-polylactide) MCL hydrogel with osteogenic factors.

To our knowledge, the functional properties of human EBs (hEBs) incorporated into *in situ*-forming hydrogels have received little attention *in vivo* [32,33,34]. Accordingly, we sought to evaluate injectable *in situ*-forming hydrogels prepared from MCL, in the context of the following specific questions (Figure 1): (a) does the *in situ*-forming MCL hydrogel act as a suitable scaffold for hEBs *in vivo*? (b) does *in vivo* osteogenic differentiation occur in response to *in situ*-forming MCL hydrogels seeded with hEBs and osteogenic factors? Resolving these questions will enhance allogeneic tissue engineering applications.

## 2. Results and Discussion

### 2.1. Preparation of an Injectable *in Situ*-Forming Hydrogel

**Figure 2:**
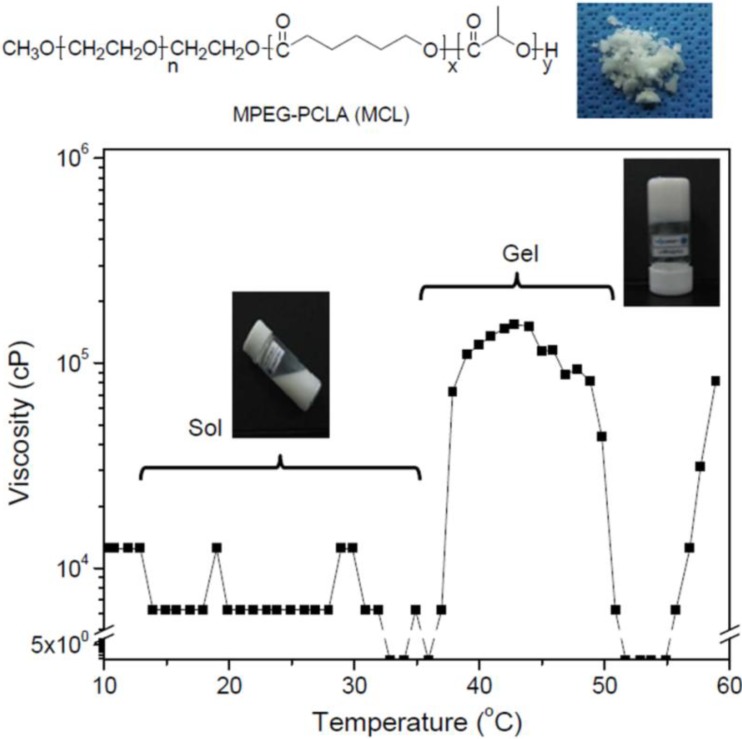
Relationships between the viscosity and temperature of the MCL solution at 20 wt % concentration.

MCL (MPEG [750 g/mol]–(polycaprolactone-*ran*-polylactide) [PCL/PLLA, 2420/90 g/mol]) was prepared using a previously reported block copolymerization method [20]. An aqueous solution of the MCL diblock copolymer was prepared in deionized water at 80 °C at a concentration of 20 wt %. As shown in Figure 2, the MCL solution existed in a translucent emulsion-solution state at room temperature. The results obtained on analyzing the phase transition as a function of temperature demonstrated that the MCL solution exhibited low viscosity below 36 °C and became a hydrogel at 37 °C.

### 2.2. *In Vivo* Gelation

**Figure 3:**
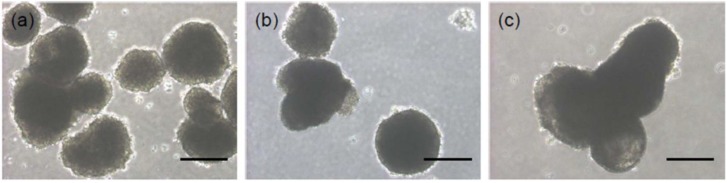
The morphology of hEBs at (**a**) 1 day; (**b**) 7 days; and (**c**) 15 days. The scale bars indicate 200 µm for the ×100 magnification.

The hESC-derived EBs were cultured in suspension for 15 days (Figure 3). On day 1, the hEBs formed a spherical mass, which is the typical morphological feature of EBs; this morphological feature was maintained for 15 days. The size of hEBs increased as the culture time increased due to the spontaneous accumulation of hEBs.

**Figure 4:**
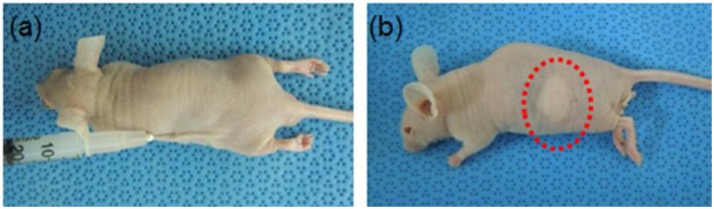
Image obtained (**a**) after the initial injection of the hEBs/F embedded in the *in situ*-forming MCL hydrogel; and (**b**) 12 weeks after hydrogel formation.

MCL containing mixtures of hEBs and osteogenic factors was easily prepared in the form of an emulsion solution. The MCL solutions containing hEBs with and without osteogenic factors were subcutaneously injected into nude mice (Figure 4a). All solutions immediately became *in situ*-forming hydrogels after injection, and the hydrogels were allowed to develop for 4, 8, and 12 weeks *in vivo*. The *in situ*-forming hydrogels maintained their shapes even after 12 weeks *in vivo* (Figure 4b). There was no inflammation at the skin surfaces of the injection sites.

**Figure 5:**
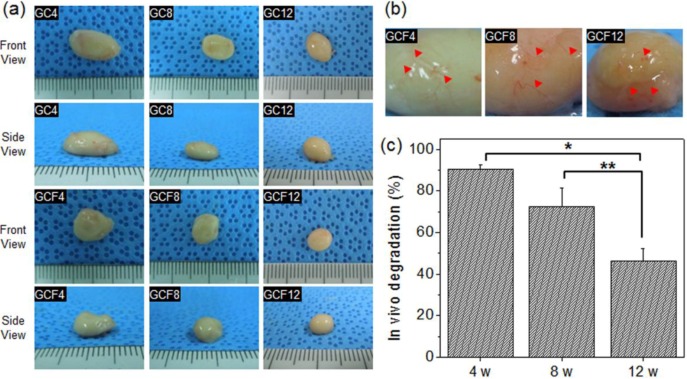
(**a**) Images of the removed MCL without (GC) or with (GCF) osteogenic factors, after 4, 8, and 12 weeks. The numbers indicate the implantation time; (**b**) enlarged images of the removed MCL hydrogels (GCF) and (**c**) volume change in the *in vivo* excised MCL hydrogels (GCF) after 4, 8, and 12 weeks (*p < 0.001, **p < 0.05).

When the hydrogels were removed after 4, 8, or 12 weeks, thin fibrous capsules containing fibroblasts and vascular vessels were observed around the surfaces of the hydrogels (Figure 5a,b). This result indicated that the *in situ*-forming MCL hydrogel acts as a suitable scaffold substrate for hEBs *in vivo*. The size of the *in situ*-forming MCL hydrogel gradually decreased after subcutaneous injection and was 90% of the original volume at 4 weeks, 72% at 8 weeks, and 47% at 12 weeks (Figure 5c). There was no large difference between GC (MCL + hEBs) and GCF (MCL + hEBs+ osteogenic factor). This finding indicated that MCL can undergo degradation in a defined experimental period as an *in situ*-forming MCL hydrogel.

### 2.3. *In Vivo* Staining

**Figure 6:**
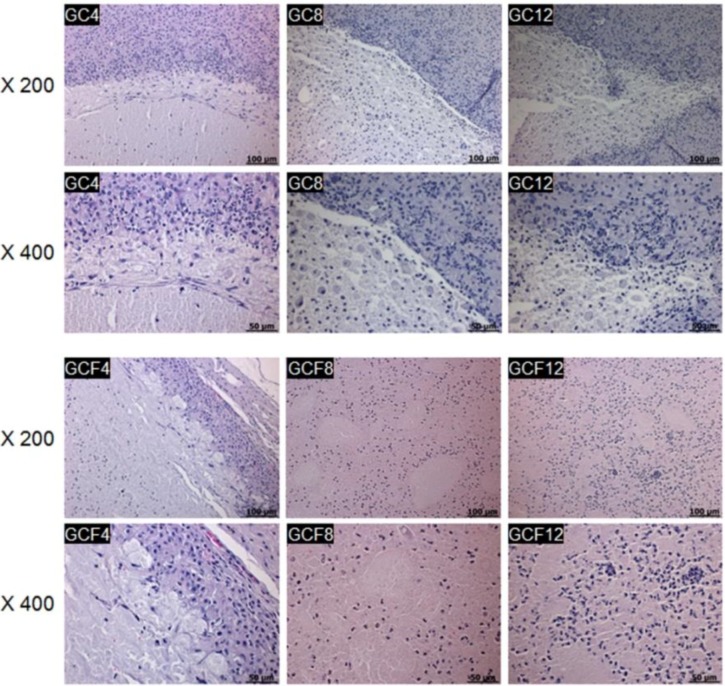
Hematoxylin and eosin (H&E) stained sections of the MCL without (GC) or with (GCF) the osteogenic factors after 4, 8, and 12 weeks. The numbers indicate the implantation time and the scale bars indicate 100 µm for the ×400 magnification and 50 µm for the ×200 magnification.

All the *in situ*-forming MCL hydrogels were stained with hematoxylin and eosin (H&E) at 4, 8, and 12 weeks after injection (Figure 6). The H&E-stained sections of the subcutaneous MCL hydrogels seeded with hEBs showed a number of cells interspersed in the *in situ*-forming MCL hydrogel. The hEBs distributed within the MCL hydrogel generally exhibited a rounded shape. The intensity of the H&E staining of the MCL hydrogel gradually decreased as the implantation time increased, probably due to the continued degradation of the MCL hydrogels.

**Figure 7:**
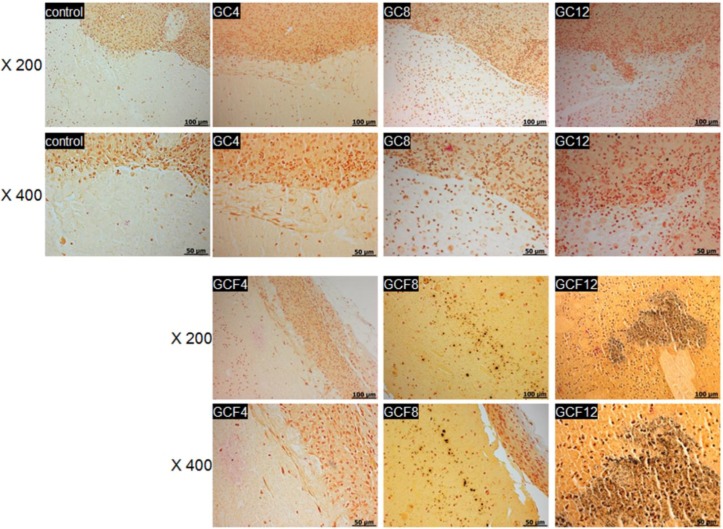
von Kossa-stained sections of the MCL without (GC) and with (GCF) the osteogenic factors after 4, 8, and 12 weeks and the MCL only without hEBs and osteogenic factors as control. The numbers indicate the implantation time and the scale bars indicate 100 µm for the ×400 magnification and 50 µm for the ×200 magnification.

After 4, 8, and 12 weeks, histological sections of the MCL hydrogels were stained using the von Kossa staining procedure to monitor mineralized bone formation associated with osteogenic differentiation of the hEBs (Figure 7). Cross-sectional control images of MCL only without hEBs and osteogenic factors showed no detectable mineral deposition. The images of von Kossa-stained hydrogels containing only hEBs without osteogenic factors also showed no evidence of mineral deposition.

In contrast, mineralized bone deposits were identified in the MCL hydrogels containing both the hEBs and osteogenic factors. The colors of the von Kossa-stained images increased as the implantation time increased, even though osteoblast differentiation could be limited, due to the complete releasing of osteogenic factors within a few days. This finding indicated that the hEBs seeded in the MCL hydrogel with the osteogenic factors had undergone differentiation toward an osteoblastic phenotype.

## 3. Experimental Section

### 3.1. Synthesis of MCL Diblock Copolymers

The block copolymerization used to produce MCL (750–2420:90; molecular ratio of PCL: PLLA) was performed as previously reported [20].

### 3.2. Viscosity Measurements

Viscosity measurements were performed using a Brookfield DV-III Ultra Viscometer equipped with a programmable thermometer and circulating baths with a programmable controller (TC-502P; Brookfield Engineering Laboratories, Middleboro, MA, USA). The viscosity of the MCL solution was determined using a T-F spindle rotating at 0.2 rpm at 10–60 °C, with temperature changes occurring in increments of 2 °C.

### 3.3. *In Vivo* Degradation of Hydrogels

For the *in vivo* biodegradation experiment, the MCL solutions were subcutaneously injected into the dorsal part of the nude mice that had been anesthetized with ethyl ether. The hydrogel implants were removed at 4, 8, and 12 weeks. The size of the MCL removed was assessed by measuring the diameter of the hydrogel in 3 dimensions with vernier calipers.

### 3.4. EB Formation

The undifferentiated human embryonic stem cell (hESC) line, SNUhES3 [35], was maintained as previously reported for research purposes, following institutional review board approval of Seoul National University Hospital and the informed consent of people undergoing *in vitro* fertilization (IVF) treatment [36]. Briefly, hESC was cultured on a mitomycin C (Sigma, St. Louis, MO, USA)-treated SIM 6-thioguanune resistant-ouabain resistant (STO) (CRL-1503; ATCC, Manassas, VA, USA) feeder layer. The hESC colonies were dissected and replated onto a fresh feeder layer every 7 days by mechanical dissociation. A mixture of DMEM/F12 (Invitrogen, CA, USA), 20% knockout serum replacement (KO-SR; Invitrogen), 1% MEM non-essential amino acids (Invitrogen, CA, USA), 0.1 mM β-mercaptoethanol (Sigma), and 4 ng/mL basic fibroblast growth factor (bFGF; Invitrogen, CA, USA) was used as the hES culture medium. In addition, the hESC culture medium was supplemented with 50 U/mL penicillin and 50 μg/mL streptomycin (Invitrogen, CA, USA).

To induce the formation of hEBs, undifferentiated hESC colonies were cultured for 5 days. Then, the hESCs were treated with collagenase type IV (5 mg/mL; Invitrogen, CA, USA). The detached hES colonies were transferred to petri dishes to allow their aggregation. The hEBs were grown in the same culture medium as the hESCs, but without bFGF. In this study, the hEBs were cultured in suspension for three and 12 days. Each EBs were collected after centrifuge and injected as actual embryoid bodies into the hydrogel.

### 3.5. *In Vivo* Experiment

Nine nude mice (weight, 20–25 g; age, 6 weeks) were divided into 2 groups of 3 mice each and used in the animal tests for 4, 8, and 12 weeks. All the animals were treated in accordance with the guidelines of the Institutional Animal Experiment Committee at the Ajou University School of Medicine. The 2 experimental groups were designated as GC (MCL + hEBs) and GCF (MCL + hEBs + osteogenic factor). The MCL was prepared as a 20 wt % solution in PBS at room temperature. The GC and GCF solutions were prepared by gently mixing all the components: a pellet containing 1 × 10^6^ hEBs, 10% FBS, 10 mM β-glyceraldehyde-3-phosphate, 60 μg L-ascorbic acid, 100 nM dexamethasone, and 150 µL of MCL solution. Within 5 min of mixing, a 150 µL syringe with a 21-gauge needle was used to inject the solution into the subcutaneous dorsum of nude mice that had been anesthetized with ethyl ether. Each nude mouse was injected with only 1 aliquot of the experimental solution. The resulting hydrogels were then allowed to develop, and biopsies were performed *in vivo* over a 4-, 8-, or 12-week period.

### 3.6. Histological Analysis

At 4, 8 and 12 weeks after implantation, the mice were sacrificed, and the implants were individually dissected and removed from the subcutaneous dorsum. The tissues were immediately fixed with 10% formalin and embedded in paraffin. The embedded specimens were sectioned (4 µm) along the longitudinal axis of the implant, and the sections were stained with H&E.

For the mineral nodule formation, the mineralized matrix was evaluated using the von Kossa stain. The sections of the hydrogel implants were washed with distilled water and then treated with 5% silver nitrate solution and kept for 60 min in a dark room. The excess silver nitrate solution was then washed away completely using distilled water, and the culture plate was treated with a sodium carbonate/formaldehyde solution for a few minutes to develop the color. Residual silver nitrate was neutralized with 5% sodium thiosulfate, and the samples were then washed 3 times with distilled water. The images were obtained using an Axio Imager A1 system (Carl Zeiss Microimaging GmbH, Göttingen, Germany) and analyzed with the Axiovision Release 4.8 software (Carl Zeiss Microimaging GmbH, Göttingen, Germany).

### 3.7. Statistical Analysis

The hydrogel implants for biodegradation were evaluated in independent experimental groups with n = 3 for each data point. All data are presented as the mean ± standard deviation (SD) values. The results were analyzed by one-way ANOVA with Bonferroni’s multiple comparison by using the SPSS software package (Version 12.0, SPSS Inc., Chicago, IL, USA).

## 4. Conclusions

Herein, we prepared an *in situ*-forming MCL hydrogel with hEBs. Our findings confirmed that the *in situ*-forming MCL hydrogel could be used as an *in vivo* substrate for hEBs and for differentiation toward an osteoblastic phenotype in nude mice. The present result suggests that hEBs seeded *in situ* by MCL hydrogels may provide numerous benefits as a noninvasive alternative for allogeneic tissue engineering applications. Further experiments are currently being performed on bone regeneration at bone-defect sites in animal models by using hEBs embedded in our *in situ*-forming MCL hydrogel. Further validations of osteogenic differentiation and mineralization are needed as future studies. In addition, further work is necessary to examine teratoma formation of hEBs.
